# Willoughby and Louisiana Medical Schools

**Published:** 1835

**Authors:** 


### IV.—Medical Department of Willoughby University, on Lake Erie; and Medical College of Louisiana, New Orleans.

It was our intention to announce to our readers the plan of organization, and the names of the professors, of these young institutions, both of which will be in their second session, with a more perfect arrangement, the ensuing winter; but, at the moment when we would refer to their circulars they are mislaid. In our next number we hope to give a more extended notice of them.

